# Supplementary figures and images for: Mobile Sleep Lab: Comparison of polysomnographic parameters with a conventional sleep laboratory
Source: PLoS One. 2025 Jan 7;20(1):e0316579. doi: 10.1371/journal.pone.0316579 (PMC11706495; doi:10.1371/journal.pone.0316579)

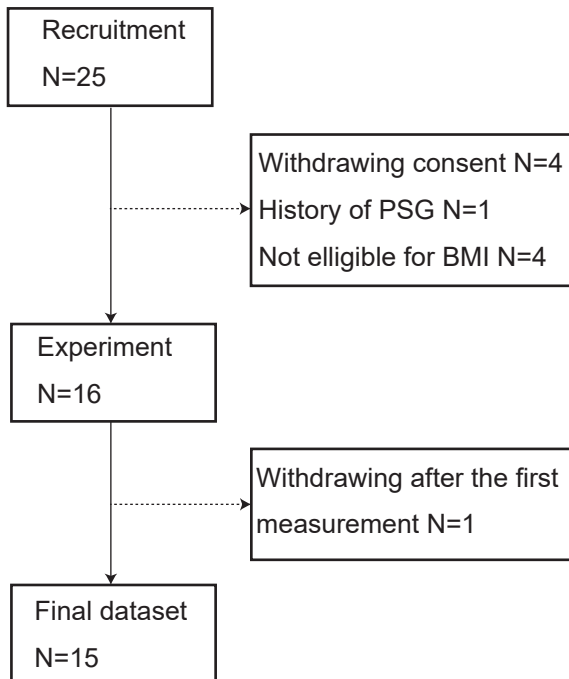

Supplement: S1 Fig — PSG, polysomnography; BMI, body mass index. (PDF) [file pone.0316579.s001.pdf]

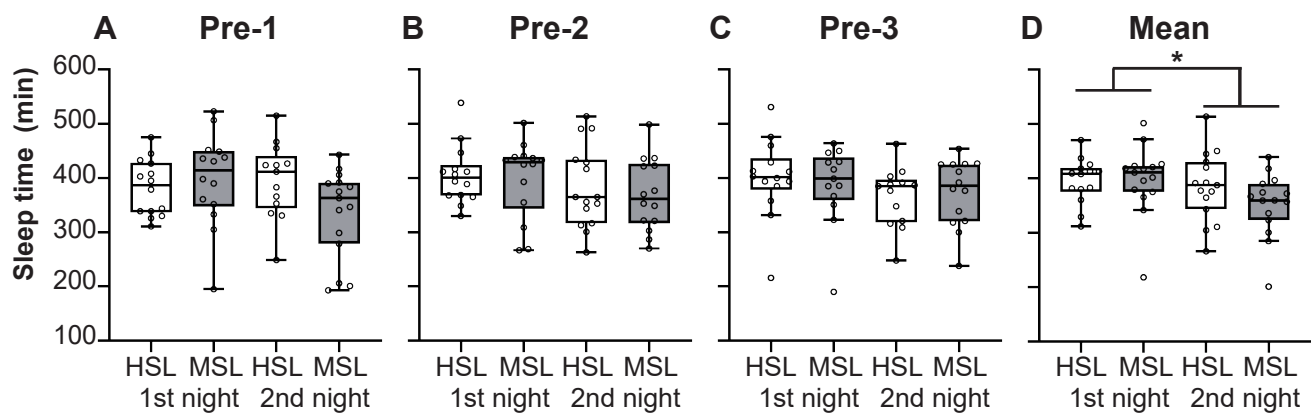

Supplement: S2 Fig — White boxes indicate the Human Sleep Lab (HSL), and gray boxes indicate the Mobile Sleep Lab (MSL). (A) pre-1: the day before the experiment, (B) pre-2: two nights before the experiment, (C) pre-3: three nights before the experiment, and (D) mean of the three nights. *p <0.05. (PDF) [file pone.0316579.s002.pdf]

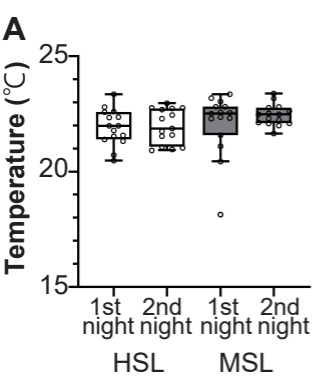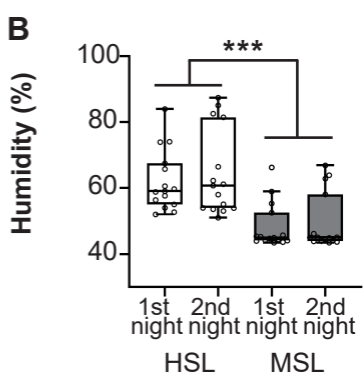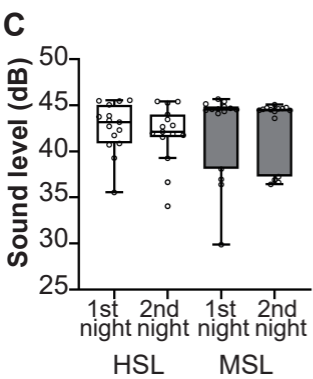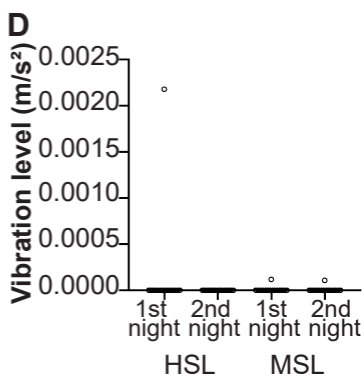

Supplement: S3 Fig — White boxes indicate the HSL, and gray boxes indicate the MSL. (A) Temperature, (B) humidity, (C) sound level, and (D) vibration level. *** p <0.001. No major seismic events were observed during the measurements that resulted in the D outlier, and the cause of this outlier remains unknown. (PDF) [file pone.0316579.s003.pdf]

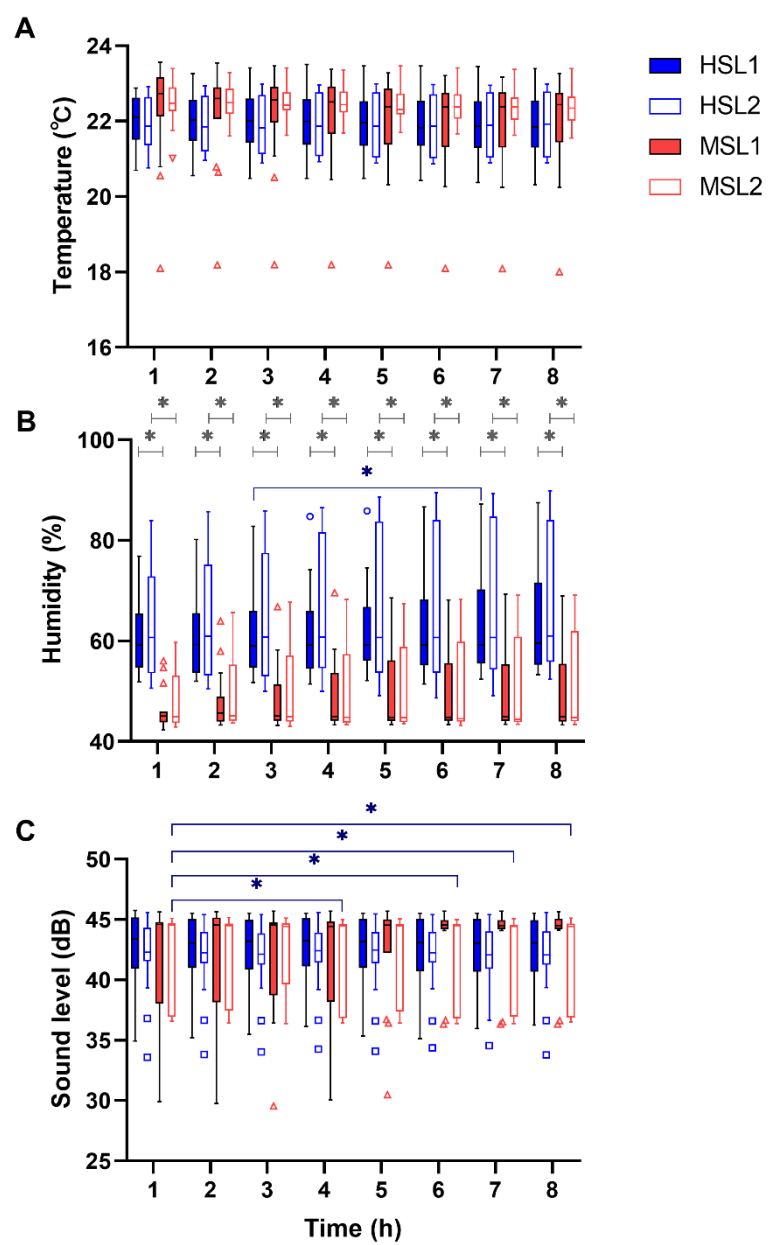

Supplement: S4 Fig — Blue boxes indicate the HSL, and red boxes indicate the MSL. (A) Temperature, (B) humidity, and (C) sound level. *p <0.05. (PDF) [file pone.0316579.s004.pdf]

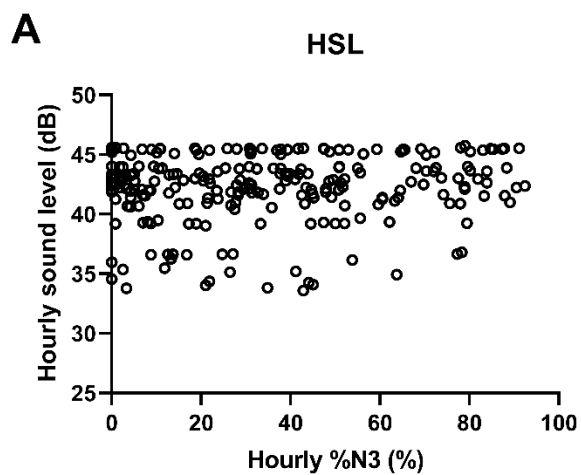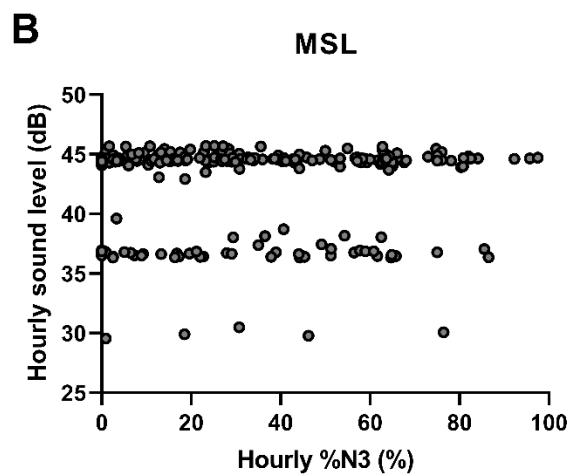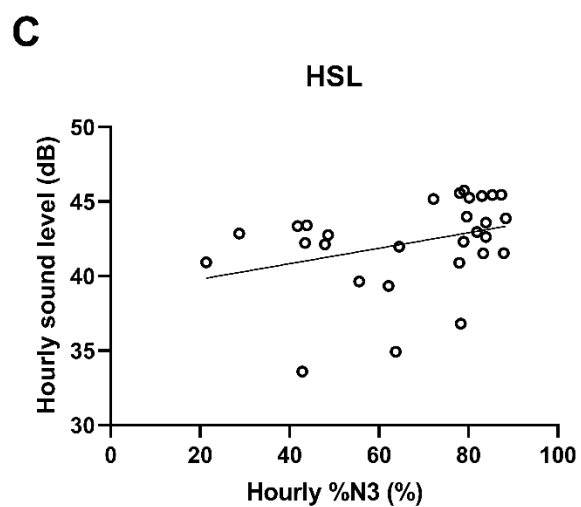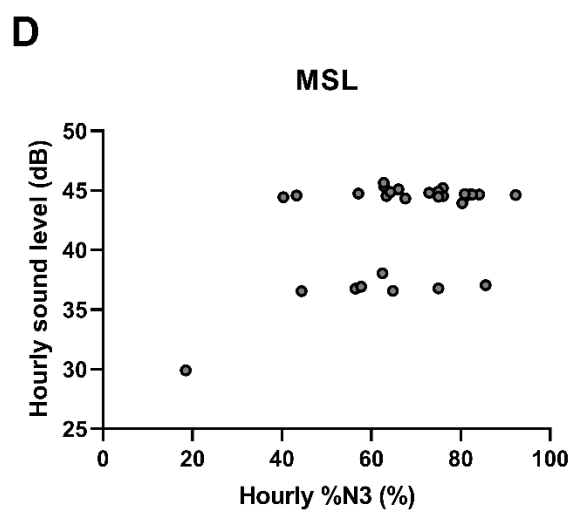

Supplement: S5 Fig — (A) The average hourly sound level and %N3 recorded in the Human Sleep Lab (HSL). (B) The average hourly sound level and %N3 recorded in the Mobile Sleep Lab (MSL). (C) The first-hour average of the sound level and %N3 recorded in the HSL. (D) The first-hour average of the sound level and %N3 recorded in the MSL. (PDF) [file pone.0316579.s005.pdf]

HSL 1st & MSL 1st night

HSL 2nd & MSL 2nd night

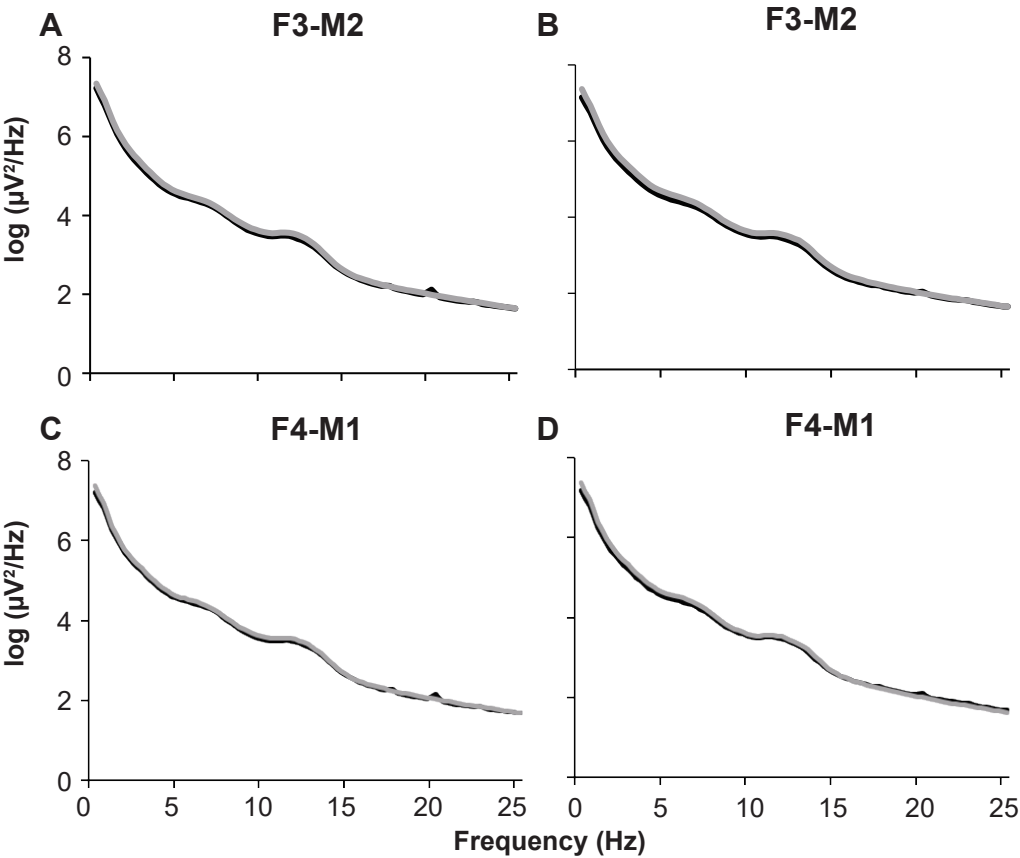

Supplement: S7 Fig — (A) F3-M2 during the first nights in the HSL and MSL, (B) F3-M2 during the second nights in the HSL and MSL, (C) F4-M1 during the first nights in the HSL and MSL, (D) F4-M1 during the second nights in the HSL and MSL. The black line indicates the HSL, and the gray line indicates the MSL. All were natural log-transformed. (PDF) [file pone.0316579.s007.pdf]
