# Supplementary material for: Mobile Sleep Lab: Comparison of polysomnographic parameters with a conventional sleep laboratory
Source: PLoS One. 2025 Jan 7;20(1):e0316579. doi: 10.1371/journal.pone.0316579 (PMC11706495; doi:10.1371/journal.pone.0316579)
Supplement: S6 Fig — (A) Stage N2 and N3 recorded at the HSL. (B) Stage N2 and N3 recorded at the MSL. (PDF) [file pone.0316579.s006.pdf]

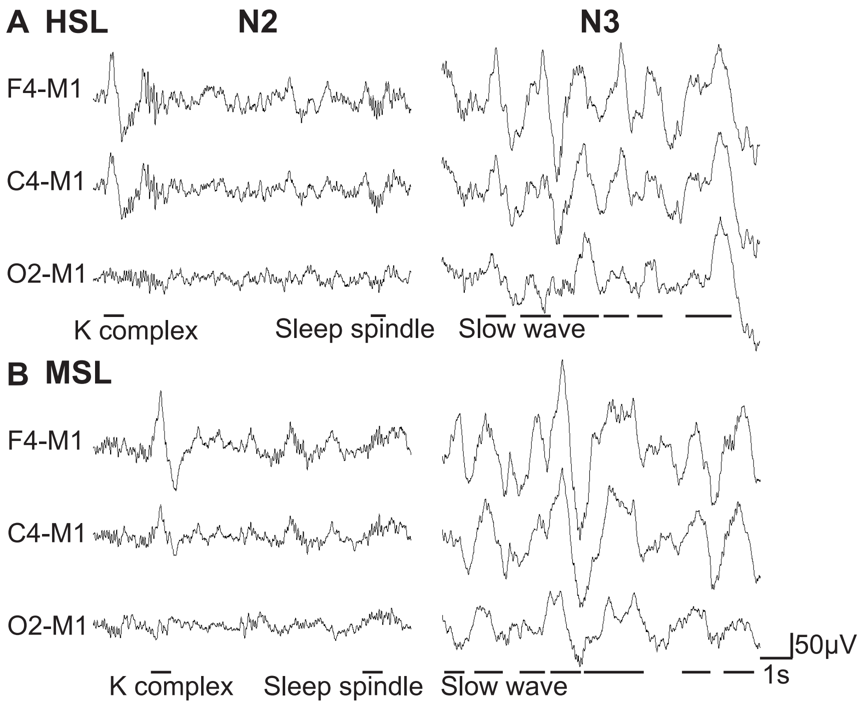
A HSL
N2
N3
F4-M1
C4-M1
O2-M1
K complex
Sleep spindle
Slow wave
B MSL
F4-M1
C4-M1
O2-M1
50μV
1s
K complex
Sleep spindle
Slow wave
