# Supplementary material for: Mobile Sleep Lab: Comparison of polysomnographic parameters with a conventional sleep laboratory
Source: PLoS One. 2025 Jan 7;20(1):e0316579. doi: 10.1371/journal.pone.0316579 (PMC11706495; doi:10.1371/journal.pone.0316579)
Supplement: S8 Fig — Comparisons between the first and second nights in the (A) HSL and (B) MSL; (C) between the first nights in the HSL and MSL; (D) between the second nights in the HSL and MSL; and (E) between the third and fourth nights in the HSL and MSL. Equivalence is determined when the 90% CI of the difference in the means of the sleep variables is within ±20% of the ratio of the difference. The black bars represent 90% CIs, the red-filled circles represent the mean of the difference between the means of the sleep variables, and the yellow regions show the range within ±20% of the ratio of the difference. ArI, arousal index; CI, confidence interval; HSL, Human Sleep Lab; MSL, Mobile Sleep Lab; SE, sleep efficiency; SL, sleep latency; TST, total sleep time, WASO, wake after sleep onset. (PDF) [file pone.0316579.s008.pdf]

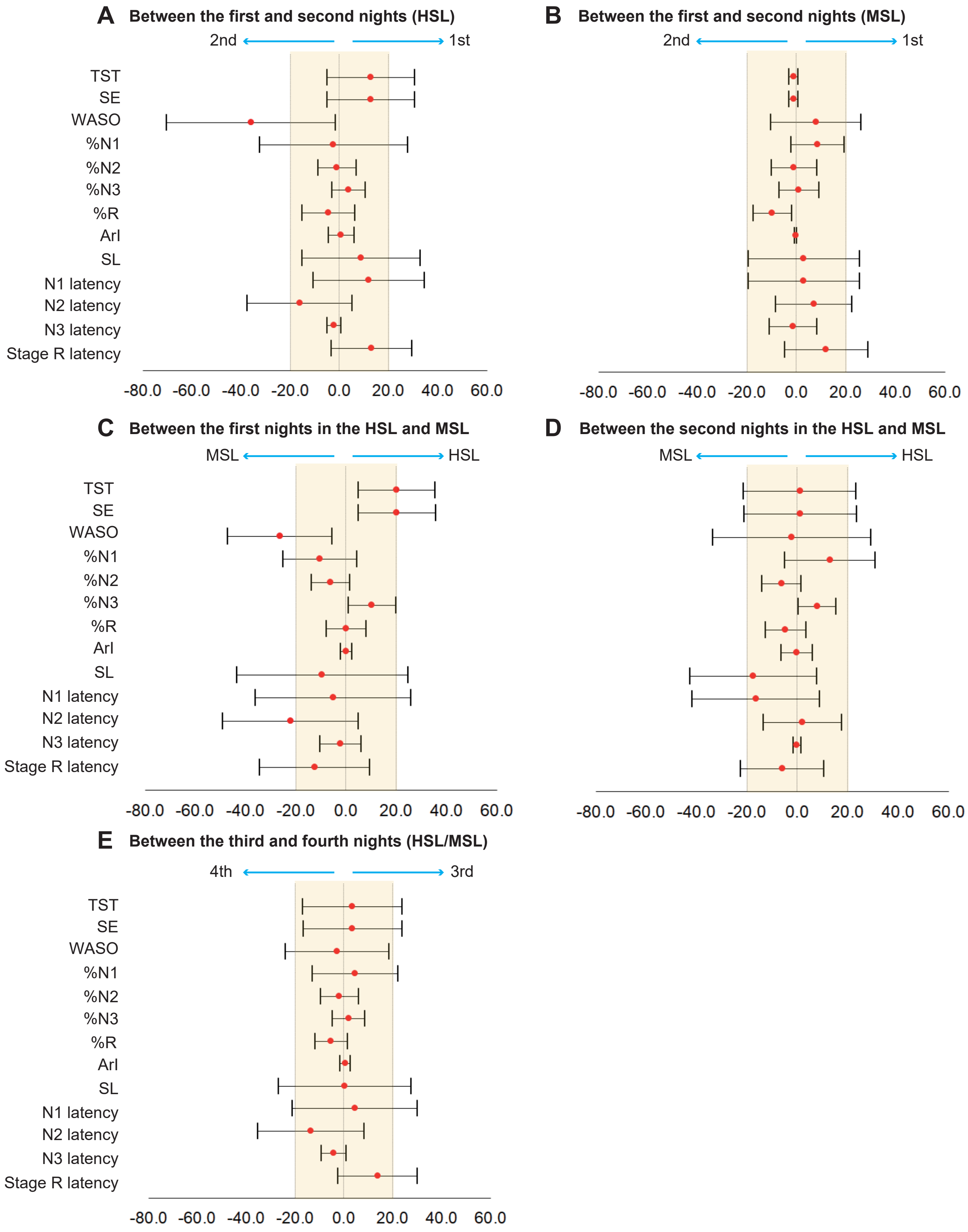
A Between the first and second nights (HSL)
2nd
1st
B Between the first and second nights (MSL)
2nd
1st
C Between the first nights in the HSL and MSL
MSL
HSL
D Between the second nights in the HSL and MSL
MSL
HSL
E Between the third and fourth nights (HSL/MSL)
4th
3rd
TST
SE
WASO
%N1
%N2
%N3
%R
ArI
SL
N1 latency
N2 latency
N3 latency
Stage R latency
-80.0
-60.0
-40.0
-20.0
0.0
20.0
40.0
60.0
