# Supplementary material for: Mobile Sleep Lab: Comparison of polysomnographic parameters with a conventional sleep laboratory
Source: PLoS One. 2025 Jan 7;20(1):e0316579. doi: 10.1371/journal.pone.0316579 (PMC11706495; doi:10.1371/journal.pone.0316579)
Supplement: S1 Table — Effect sizes (Cohen’s d) of the variables without an asterisk (*) were calculated using least square means and standard error from the linear mixed model. Effect sizes with an asterisk (*) were calculated using raw data. ArI, arousal index; HSL, Human Sleep Lab; MSL, Mobile Sleep Lab; SE, sleep efficiency; SL, sleep latency; TST, total sleep time; WASO, wake after sleep onset; OSA-MA, Oguri-Shirakawa-Azumi Sleep Inventory, Middle-age and Aged version. (DOCX) [file pone.0316579.s009.docx]

**S1 Table. Effect sizes of the parameters.**

| **Parameter** | **Between the HSL and MSL** | **Between the HSL and MSL during the first night** | **Between the HSL and MSL during the second night** | **Between the first and second nights in the HSL** | **Between the first and second nights in the MSL** |
| --- | --- | --- | --- | --- | --- |
| **TST** | 0.37 | 0.61 | 0.03 | 0.35 | 0.23 |
| **SL** | 0.25 | 0.15 | 0.28 | 0.18 | 0.05 |
| **SE** | 0.37 | 0.61 | 0.03 | 0.35 | 0.23 |
| **WASO** | 0.38 | 0.69 | 0.07 | 0.52 | 0.24 |
| **%N1** | 0.12 | 0.35 | 0.14 | 0.13 | 0.35 |
| **%N2** | 0.35 | 0.31 | 0.32 | 0.04 | 0.05 |
| **%N3** | 0.45 | 0.47 | 0.35 | 0.17 | 0.05 |
| **%R** | 0.13 | 0.03 | 0.24 | 0.23 | 0.50 |
| **N1 latency** | 0.22 | 0.08 | 0.28 | 0.25 | 0.04 |
| **N2 latency** | 0.20 | 0.46 | 0.12 | 0.38 | 0.19 |
| **N3 latency** | 0.05 | 0.14 | 0.05 | 0.35 | 0.16 |
| **Stage R latency^*^** | 0.26 | 0.31 | 0.21 | 0.37 | 0.33 |
| **ArI** | 0.02 | 0.05 | 0.08 | 0.05 | 0.07 |
|  |  |  |  |  |  |
| **OSA-MA  “Sleepiness on rising” ^*^** | 0.37 | 0.27 | 0.47 | 0.22 | 0.06 |
| **OSA-MA  “Initiation and maintenance of sleep”** | 0.84 | 0.94 | 0.34 | 0.07 | 0.53 |
| **OSA-MA**  **“Frequent dreaming, nightmares” ^*^** | 0.21 | 0.08 | 0.34 | 0.18 | 0.09 |
| **OSA-MA  “Refreshness” ^*^** | 0.23 | 0.05 | 0.38 | 0.21 | 0.15 |
| **OSA-MA  “Sleep duration” ^*^** | 0.07 | 0.06 | 0.21 | 0.05 | 0.24 |
| **Temperature** | 0.63 | 0.39 | 0.45 | 0.03 | 0.09 |
| **Humidity^*^** | 1.51 | 1.71 | 1.35 | 0.20 | 0.16 |
| **Sound level^*^** | 0.04 | 0.08 | 0.18 | 0.24 | 0.06 |
| **Vibration level^*^** | 0.23 | 0.34 | 0.37 | 0.37 | 0.02 |
| Effect sizes (Cohen’s *d*) of the variables without an asterisk (*) were calculated using least square means and standard error from the linear mixed model. Effect sizes with an asterisk (*) were calculated using raw data.  ArI, arousal index; HSL, Human Sleep Lab; MSL, Mobile Sleep Lab; SE, sleep efficiency; SL, sleep latency; TST, total sleep time; WASO, wake after sleep onset; OSA-MA, Oguri-Shirakawa-Azumi Sleep Inventory, Middle-age and Aged version. | | | | | |
