# Supplementary material for: Mobile Sleep Lab: Comparison of polysomnographic parameters with a conventional sleep laboratory
Source: PLoS One. 2025 Jan 7;20(1):e0316579. doi: 10.1371/journal.pone.0316579 (PMC11706495; doi:10.1371/journal.pone.0316579)
Supplement: S2 Table — Order indicates the place (HSL or MSL) on the first and second nights and the third and fourth nights. Pre-exp represents on which night before the experiment the measurement was carried out. Place compares the HSL and MSL. Time compares the first nights (HSL and MSL) and the second nights (HSL and MSL). HSL, Human Sleep Lab; MSL, Mobile Sleep Lab. (DOCX) [file pone.0316579.s010.docx]

**S2 Table. Results of statistical analyses of sleep time obtained from Fitbit Charge 3 based on order, pre-exp, place, and time**.

| **Parameters** | **Transformation** | **Covariance structures** | **Effect** | ***F*-value** | ***P*-value** |
| --- | --- | --- | --- | --- | --- |
| **Fitbit_Sleep time** | Box-Cox | Compound Symmetry | Order | *F*_1, 12.8_ = 2.27 | 0.1559 |
|  |  |  | Pre-exp | *F*_2, 142_ = 0.31 | 0.7321 |
|  |  |  | Place | *F*_1, 142_ = 1.31 | 0.2541 |
|  |  |  | Time | *F*_1, 141_ = 6.53 | 0.0117 |
|  |  |  | Pre-exp×Place | *F*_2, 141_ = 0.27 | 0.7638 |
|  |  |  | Pre-exp×Time | *F*_2, 142_ = 0.01 | 0.9903 |
|  |  |  | Place×Time | *F*_1, 142_ = 1.01 | 0.3176 |
|  |  |  | Pre-exp×Place×Time | *F*_2, 143_ = 2.68 | 0.0724 |
| Order indicates the Place (HSL or MSL) on the first and second nights and the third and fourth nights. Pre-exp represents on which night before the experiment the measurement was carried out. Place compares the HSL and MSL. Time compares the first nights (HSL and MSL) and the second nights (HSL and MSL).  HSL, Human Sleep Lab; MSL, Mobile Sleep Lab | | | | | |
|  |  |  |  |  |  |
