# Supplementary material for: Mobile Sleep Lab: Comparison of polysomnographic parameters with a conventional sleep laboratory
Source: PLoS One. 2025 Jan 7;20(1):e0316579. doi: 10.1371/journal.pone.0316579 (PMC11706495; doi:10.1371/journal.pone.0316579)
Supplement: S3 Table — Order indicates the place (HSL or MSL) on the first and second nights and the third and fourth nights. Pre-exp represents on which night before the experiment the measurement was carried out. Place compares the HSL and MSL. Time compares the first nights (HSL and MSL) and the second nights (HSL and MSL). ArI, arousal index; CV, coefficient of variation; HSL, Human Sleep Lab; MSL, Mobile Sleep Lab; SE, sleep efficiency; SL, sleep latency; SWA, slow-wave activity; TST, total sleep time; WASO, wake after sleep onset; OSA-MA, Oguri-Shirakawa-Azumi Sleep Inventory, Middle-age and Aged version. (DOCX) [file pone.0316579.s011.docx]

**S3 Table. Results of statistical analyses of sleep parameters, self-reported sleep quality, temperature, and coefficient of variation of environmental factors based on order, place, and time using a linear mixed model**.

| **Parameter** | **Transformation** | **Covariance structures** | **Effect** | ***F*-value** | ***P*-value** |
| --- | --- | --- | --- | --- | --- |
| **TST** | Box-Cox | Compound Symmetry | Order | *F*_1, 13_ = 1.44 | 0.2512 |
|  |  |  | Place | *F*_1, 42_ = 2.79 | 0.1025 |
|  |  |  | Time | *F*_1, 42_ = 0.10 | 0.7496 |
|  |  |  | Place×Time | *F*_1, 42_ = 2.31 | 0.1358 |
| **SE** | Box-Cox | Compound Symmetry | Order | *F*_1, 13_ = 1.46 | 0.2490 |
|  |  |  | Place | *F*_1, 42_ = 2.80 | 0.1018 |
|  |  |  | Time | *F*_1, 42_ = 0.11 | 0.7463 |
|  |  |  | Place×Time | *F*_1, 42_ = 2.30 | 0.1369 |
| **SL** | Box-Cox | Autoregressive | Order | *F*_1, 14.2_ = 1.46 | 0.2474 |
|  |  |  | Place | *F*_1, 52.6_ = 0.74 | 0.3931 |
|  |  |  | Time | *F*_1, 29_ = 0.45 | 0.5071 |
|  |  |  | Place×Time | *F*_1, 37.8_ = 0.10 | 0.7556 |
| **WASO** | Box-Cox | Compound Symmetry | Order | *F*_1, 13_ = 1.25 | 0.2833 |
|  |  |  | Place | *F*_1, 42_ = 2.22 | 0.1434 |
|  |  |  | Time | *F*_1, 42_ = 0.46 | 0.5012 |
|  |  |  | Place×Time | *F*_1, 42_ = 3.36 | 0.0739 |
| **%N1** | Box-Cox | Compound Symmetry | Order | *F*_1, 13_ = 0.04 | 0.8429 |
|  |  |  | Place | *F*_1, 42_ = 0.31 | 0.5819 |
|  |  |  | Time | *F*_1, 42_ = 0.34 | 0.5650 |
|  |  |  | Place×Time | *F*_1, 42_ = 1.65 | 0.2058 |
| **%N2** | Raw data | Compound Symmetry | Order | *F*_1, 13_ = 1.12 | 0.3093 |
|  |  |  | Place | *F*_1, 42_ = 3.80 | 0.0579 |
|  |  |  | Time | *F*_1, 42_ = 0.07 | 0.7860 |
|  |  |  | Place×Time | *F*_1, 42_ = 0.00 | 0.9895 |
| **%N3** | Raw data | Compound Symmetry | Order | *F*_1, 13_ = 0.26 | 0.6171 |
|  |  |  | Place | *F*_1, 42_ = 8.62 | 0.0054 |
|  |  |  | Time | *F*_1, 42_ = 0.61 | 0.4409 |
|  |  |  | Place×Time | *F*_1, 42_ = 0.20 | 0.6552 |
| **%R** | Raw data | Compound Symmetry | Order | *F*_1, 13_ = 0.53 | 0.4783 |
|  |  |  | Place | *F*_1, 42_ = 0.35 | 0.5593 |
|  |  |  | Time | *F*_1, 42_ = 3.89 | 0.0553 |
|  |  |  | Place×Time | *F*_1, 42_ = 0.53 | 0.4702 |
| **N1 latency** | Box-Cox | Compound Symmetry | Order | *F*_1, 13_ = 1.21 | 0.2913 |
|  |  |  | Place | *F*_1, 42_ = 0.85 | 0.3620 |
|  |  |  | Time | *F*_1, 42_ = 0.54 | 0.4650 |
|  |  |  | Place×Time | *F*_1, 42_ = 0.26 | 0.6098 |
| **N2 latency** | Box-Cox | Compound Symmetry | Order | *F*_1, 13_ = 1.00 | 0.3351 |
|  |  |  | Place | *F*_1, 42_ = 0.80 | 0.3759 |
|  |  |  | Time | *F*_1, 42_ = 0.26 | 0.6128 |
|  |  |  | Place×Time | *F*_1, 42_ = 2.27 | 0.1390 |
| **N3 latency** | Box-Cox | Compound Symmetry | Order | *F*_1, 13_ = 0.00 | 0.9918 |
|  |  |  | Place | *F*_1, 42_ = 0.05 | 0.8171 |
|  |  |  | Time | *F*_1, 42_ = 1.90 | 0.1758 |
|  |  |  | Place×Time | *F*_1, 42_ = 0.25 | 0.6205 |
| **ArI** | Box-Cox | Compound Symmetry | Order | *F*_1, 13_ = 0.61 | 0.4489 |
|  |  |  | Place | *F*_1, 42_ = 0.01 | 0.9349 |
|  |  |  | Time | *F*_1, 42_ = 0.00 | 0.9664 |
|  |  |  | Place×Time | *F*_1, 42_ = 0.10 | 0.7558 |
| **SWA-F3 during N2 and N3** | Raw data | Compound Symmetry | Order | *F*_1, 13_ = 0.23 | 0.6369 |
|  |  |  | Place | *F*_1, 42_ *=* 0.06 | 0.8100 |
|  |  |  | Time | *F*_1, 42_ *=* 0.01 | 0.9041 |
|  |  |  | Place×Time | *F*_1, 42_ *=* 0.00 | 0.9871 |
| **SWA-F4 during N2 and N3** | Raw data | Compound Symmetry | Order | *F*_1, 13_ = 0.08 | 0.7803 |
|  |  |  | Place | *F*_1, 42_ = 0.13 | 0.7180 |
|  |  |  | Time | *F*_1, 42_ = 0.06 | 0.8071 |
|  |  |  | Place×Time | *F*_1, 42_ = 0.23 | 0.6348 |
| **SWA-C3 during N2 and N3** | Raw data | Compound Symmetry | Order | *F*_1, 13_ = 0.24 | 0.6320 |
|  |  |  | Place | *F*_1, 42_ = 0.23 | 0.6314 |
|  |  |  | Time | *F*_1, 42_ = 0.36 | 0.5545 |
|  |  |  | Place×Time | *F*_1, 42_ = 0.46 | 0.5033 |
| **SWA-C4 during N2 and N3** | Box-Cox | Compound Symmetry | Order | *F*_1, 13_ = 0.01 | 0.9326 |
|  |  |  | Place | *F*_1, 42_ = 2.79 | 0.1024 |
|  |  |  | Time | *F*_1, 42_ = 0.11 | 0.7441 |
|  |  |  | Place×Time | *F*_1, 42_ = 0.33 | 0.5700 |
| **SWA-O1 during N2 and N3** | Box-Cox | Compound Symmetry | Order | *F*_1, 13_ = 0.53 | 0.4813 |
|  |  |  | Place | *F*_1, 42_ = 2.05 | 0.1592 |
|  |  |  | Time | *F*_1, 42_ = 0.10 | 0.7558 |
|  |  |  | Place×Time | *F*_1, 42_ = 0.31 | 0.5817 |
| **SWA-O2 during N2 and N3** | Box-Cox | Unstructured | Order | *F*_1, 13.8_ = 1.86 | 0.1950 |
|  |  |  | Place | *F*_1, 13.6_ = 0.19 | 0.6662 |
|  |  |  | Time | *F*_1, 14_ = 0.36 | 0.5559 |
|  |  |  | Place×Time | *F*_1, 14_ = 0.27 | 0.6127 |
| **SWE-F3 during N2 and N3** | Box-Cox | Compound Symmetry | Order | *F*_1, 13_ = 0.00 | 0.9802 |
|  |  |  | Place | *F*_1, 42_ = 0.53 | 0.4693 |
|  |  |  | Time | *F*_1, 42_ = 0.10 | 0.7490 |
|  |  |  | Place×Time | *F*_1, 42_ = 0.28 | 0.5968 |
| **SWE-F4 during N2 and N3** | Box-Cox | Compound Symmetry | Order | *F*_1, 13_ = 0.00 | 0.9589 |
|  |  |  | Place | *F*_1, 42_ = 0.57 | 0.4539 |
|  |  |  | Time | *F*_1, 42_ = 0.08 | 0.7816 |
|  |  |  | Place×Time | *F*_1, 42_ = 0.29 | 0.5956 |
| **SWE-C3 during N2 and N3** | Box-Cox | Compound Symmetry | Order | *F*_1, 13_ = 0.03 | 0.8580 |
|  |  |  | Place | *F*_1, 42_ = 0.69 | 0.4095 |
|  |  |  | Time | *F*_1, 42_ = 0.20 | 0.6544 |
|  |  |  | Place×Time | *F*_1, 42_ = 0.27 | 0.6080 |
| **SWE-C4 during N2 and N3** | Box-Cox | Compound Symmetry | Order | *F*_1, 13_ = 0.01 | 0.9177 |
|  |  |  | Place | *F*_1, 42_ = 0.30 | 0.5848 |
|  |  |  | Time | *F*_1, 42_ = 0.15 | 0.7028 |
|  |  |  | Place×Time | *F*_1, 42_ = 0.53 | 0.4700 |
| **SWE-O1 during N2 and N3** | Box-Cox | Compound Symmetry | Order | *F*_1, 13_ = 0.05 | 0.8233 |
|  |  |  | Place | *F*_1, 42_ = 0.50 | 0.4849 |
|  |  |  | Time | *F*_1, 42_ = 0.16 | 0.6901 |
|  |  |  | Place×Time | *F*_1, 42_ = 0.18 | 0.6731 |
| **SWE-O2 during N2 and N3** | Box-Cox | Compound Symmetry | Order | *F*_1, 13_ = 0.09 | 0.7745 |
|  |  |  | Place | *F*_1, 42_ *=* 0.80 | 0.3752 |
|  |  |  | Time | *F*_1, 42_ *=* 0.07 | 0.7963 |
|  |  |  | Place×Time | *F*_1, 42_ *=* 0.38 | 0.5409 |
| **Delta-F3 during N2 and N3** | Box-Cox | Compound Symmetry | Order | *F*_1, 13_ = 0.06 | 0.8082 |
|  |  |  | Place | *F*_1, 42_ = 0.26 | 0.6145 |
|  |  |  | Time | *F*_1, 42_ = 0.25 | 0.6214 |
|  |  |  | Place×Time | *F*_1, 42_ = 0.21 | 0.6465 |
| **Delta-F4 during N2 and N3** | Box-Cox | Compound Symmetry | Order | *F*_1, 13_ = 0.00 | 0.9527 |
|  |  |  | Place | *F*_1, 42_ = 0.24 | 0.6257 |
|  |  |  | Time | *F*_1, 42_ = 0.35 | 0.5566 |
|  |  |  | Place×Time | *F*_1, 42_ = 0.05 | 0.8215 |
| **Delta-C3 during N2 and N3** | Raw data | Compound Symmetry | Order | *F*_1, 13_ = 0.11 | 0.7403 |
|  |  |  | Place | *F*_1, 42_ = 0.66 | 0.4224 |
|  |  |  | Time | *F*_1, 42_ = 0.00 | 0.9643 |
|  |  |  | Place×Time | *F*_1, 42_ = 0.34 | 0.5607 |
| **Delta-C4 during N2 and N3** | Box-Cox | Compound Symmetry | Order | *F*_1, 13_ = 0.14 | 0.7121 |
|  |  |  | Place | *F*_1, 42_ = 4.80 | 0.0341 |
|  |  |  | Time | *F*_1, 42_ = 0.21 | 0.6519 |
|  |  |  | Place×Time | *F*_1, 42_ = 0.22 | 0.6393 |
| **Delta-O1 during N2 and N3** | Box-Cox | Compound Symmetry | Order | *F*_1, 13_ = 0.29 | 0.5964 |
|  |  |  | Place | *F*_1, 42_ = 2.19 | 0.1466 |
|  |  |  | Time | *F*_1, 42_ = 0.00 | 0.9608 |
|  |  |  | Place×Time | *F*_1, 42_ = 0.10 | 0.7481 |
| **Delta-O2 during N2 and N3** | Raw data | Unstructured | Order | *F*_1, 13.6_ = 1.08 | 0.3169 |
|  |  |  | Place | *F*_1, 13.5_ = 0.00 | 0.9685 |
|  |  |  | Time | *F*_1, 14_ = 0.39 | 0.5447 |
|  |  |  | Place×Time | *F*_1, 14_ = 0.02 | 0.8900 |
| **SO-F3 during N2 and N3** | Raw data | Compound Symmetry | Order | *F*_1, 13_ = 0.60 | 0.4507 |
|  |  |  | Place | *F*_1, 42_ = 0.23 | 0.6365 |
|  |  |  | Time | *F*_1, 42_ = 0.07 | 0.7984 |
|  |  |  | Place×Time | *F*_1, 42_ = 0.03 | 0.8532 |
| **SO-F4 during N2 and N3** | Raw data | Compound Symmetry | Order | *F*_1, 13_ = 0.33 | 0.5731 |
|  |  |  | Place | *F*_1, 42_ = 0.03 | 0.8655 |
|  |  |  | Time | *F*_1, 42_ = 0.01 | 0.9269 |
|  |  |  | Place×Time | *F*_1, 42_ = 0.43 | 0.5157 |
| **SO-C3 during N2 and N3** | Raw data | Compound Symmetry | Order | *F*_1, 13_ = 0.09 | 0.7726 |
|  |  |  | Place | *F*_1, 42_ = 0.11 | 0.7365 |
|  |  |  | Time | *F*_1, 42_ = 0.74 | 0.3949 |
|  |  |  | Place×Time | *F*_1, 42_ = 0.40 | 0.5289 |
| **SO-C4 during N2 and N3** | Box Cox | Compound Symmetry | Order | *F*_1, 13_ = 0.22 | 0.6464 |
|  |  |  | Place | *F*_1, 42_ = 1.92 | 0.1733 |
|  |  |  | Time | *F*_1, 42_ = 0.01 | 0.9413 |
|  |  |  | Place×Time | *F*_1, 42_ = 0.29 | 0.5926 |
| **SO-O1 during N2 and N3** | Box Cox | Compound Symmetry | Order | *F*_1, 13_ = 0.26 | 0.6211 |
|  |  |  | Place | *F*_1, 42_ = 2.01 | 0.1637 |
|  |  |  | Time | *F*_1, 42_ = 0.32 | 0.5722 |
|  |  |  | Place×Time | *F*_1, 42_ = 0.37 | 0.5447 |
| **SO-O2 during N2 and N3** | Box Cox | Unstructured | Order | *F*_1, 13.7_ = 1.04 | 0.3259 |
|  |  |  | Place | *F*_1, 13.8_ = 0.48 | 0.5018 |
|  |  |  | Time | *F*_1, 14_ = 0.17 | 0.6854 |
|  |  |  | Place×Time | *F*_1, 14_ = 0.37 | 0.5536 |
| **Theta-F4 during N2 and N3** | Raw data | Compound Symmetry | Order | *F*_1, 13_ = 0.87 | 0.3668 |
|  |  |  | Place | *F*_1, 42_ = 0.71 | 0.4044 |
|  |  |  | Time | *F*_1, 42_ = 0.18 | 0.6731 |
|  |  |  | Place×Time | *F*_1, 42_ = 0.00 | 0.9652 |
| **Theta-C4 during N2 and N3** | Raw data | Unstructured | Order | *F*_1, 11.6_ = 1.43 | 0.2561 |
|  |  |  | Place | *F*_1, 13.4_ = 4.51 | 0.0528 |
|  |  |  | Time | *F*_1, 12.4_ *=* 1.00 | 0.3366 |
|  |  |  | Place×Time | *F*_1, 13.6_ = 0.03 | 0.8694 |
| **Theta-O1 during N2 and N3** | Raw data | Compound Symmetry | Order | *F*_1, 13_ = 0.01 | 0.9194 |
|  |  |  | Place | *F*_1, 42_ = 1.58 | 0.2151 |
|  |  |  | Time | *F*_1, 42_ = 0.00 | 0.9675 |
|  |  |  | Place×Time | *F*_1, 42_ = 1.09 | 0.3014 |
| **Theta-O2 during N2 and N3** | Raw data | Compound Symmetry | Order | *F*_1, 13_ = 0.11 | 0.7465 |
|  |  |  | Place | *F*_1, 42_ = 0.12 | 0.7275 |
|  |  |  | Time | *F*_1, 42_ = 0.06 | 0.8020 |
|  |  |  | Place×Time | *F*_1, 42_ = 0.03 | 0.8678 |
| **Alpha-F3 during N2 and N3** | Raw data | Compound Symmetry | Order | *F*_1, 13_ = 0.05 | 0.8195 |
|  |  |  | Place | *F*_1, 42_ = 0.09 | 0.7612 |
|  |  |  | Time | *F*_1, 42_ = 3.16 | 0.0827 |
|  |  |  | Place×Time | *F*_1, 42_ = 0.18 | 0.6776 |
| **Alpha-F4 during N2 and N3** | Box-Cox | Compound Symmetry | Order | *F*_1, 13_ = 0.16 | 0.6965 |
|  |  |  | Place | *F*_1, 42_ = 1.16 | 0.2886 |
|  |  |  | Time | *F*_1, 42_ = 0.00 | 0.9985 |
|  |  |  | Place×Time | *F*_1, 42_ = 0.21 | 0.6467 |
| **Alpha-C3 during N2 and N3** | Box-Cox | Autoregressive | Order | *F*_1, 13.5_ = 0.12 | 0.7328 |
|  |  |  | Place | *F*_1, 42.4_ *=* 0.06 | 0.8138 |
|  |  |  | Time | *F*_1, 40_ *=* 1.84 | 0.1823 |
|  |  |  | Place×Time | *F*_1, 39.6_ *=* 0.72 | 0.4004 |
| **Alpha-C4 during N2 and N3** | Raw data | Unstructured | Order | *F*_1, 13_ *=* 0.01 | 0.9386 |
|  |  |  | Place | *F*_1, 13.7_ *=* 6.89 | 0.0203 |
|  |  |  | Time | *F*_1, 12.9_ *=* 5.42 | 0.0368 |
|  |  |  | Place×Time | *F*_1, 13.5_ *=* 0.01 | 0.9238 |
| **Alpha-O1 during N2 and N3** | Raw data | Compound Symmetry | Order | *F*_1, 13_ = 0.16 | 0.6939 |
|  |  |  | Place | *F*_1, 42_ = 2.41 | 0.1281 |
|  |  |  | Time | *F*_1, 42_ = 0.04 | 0.8412 |
|  |  |  | Place×Time | *F*_1, 42_ = 0.53 | 0.4700 |
| **Alpha-O2 during N2 and N3** | Raw data | Compound Symmetry | Order | *F*_1, 13_ = 0.78 | 0.3937 |
|  |  |  | Place | *F*_1, 42_ = 0.16 | 0.6942 |
|  |  |  | Time | *F*_1, 42_ = 0.00 | 0.9859 |
|  |  |  | Place×Time | *F*_1, 42_ = 0.01 | 0.9283 |
| **Sigma-F3 during N2 and N3** | Raw data | Compound Symmetry | Order | *F*_1, 13_ = 1.97 | 0.1834 |
|  |  |  | Place | *F*_1, 42_ = 0.37 | 0.5455 |
|  |  |  | Time | *F*_1, 42_ = 3.71 | 0.0608 |
|  |  |  | Place×Time | *F*_1, 42_ = 0.00 | 0.9564 |
| **Sigma-F4 during N2 and N3** | Raw data | Compound Symmetry | Order | *F*_1, 13_ = 2.94 | 0.1104 |
|  |  |  | Place | *F*_1, 42_ = 1.10 | 0.2992 |
|  |  |  | Time | *F*_1, 42_ = 0.37 | 0.5445 |
|  |  |  | Place×Time | *F*_1, 42_ = 0.02 | 0.8825 |
| **Sigma-C3 during N2 and N3** | Box-Cox | Compound Symmetry | Order | *F*_1, 13_ = 2.56 | 0.1335 |
|  |  |  | Place | *F*_1, 42_ = 0.97 | 0.3296 |
|  |  |  | Time | *F*_1, 42_ = 0.71 | 0.4049 |
|  |  |  | Place×Time | *F*_1, 42_ = 1.41 | 0.2410 |
| **Sigma-C4 during N2 and N3** | Raw data | Unstructured | Order | *F*_1, 13_ *=* 2.05 | 0.1757 |
|  |  |  | Place | *F*_1, 13.9_ *=* 11.26 | 0.0048 |
|  |  |  | Time | *F*_1, 13.4_ *=* 7.93 | 0.0143 |
|  |  |  | Place×Time | *F*_1, 13.4_ *=* 0.00 | 0.9458 |
| **Sigma-O1 during N2 and N3** | Raw data | Compound Symmetry | Order | *F*_1, 13_ = 1.69 | 0.2166 |
|  |  |  | Place | *F*_1, 42_ = 2.46 | 0.1240 |
|  |  |  | Time | *F*_1, 42_ = 0.01 | 0.9373 |
|  |  |  | Place×Time | *F*_1, 42_ = 0.61 | 0.4380 |
| **Sigma-O2 during N2 and N3** | Raw data | Compound Symmetry | Order | *F*_1, 13_ = 3.35 | 0.0901 |
|  |  |  | Place | *F*_1, 42_ = 0.30 | 0.5844 |
|  |  |  | Time | *F*_1, 42_ = 0.00 | 0.9543 |
|  |  |  | Place×Time | *F*_1, 42_ = 0.02 | 0.9009 |
| **Beta-F3 during N2 and N3** | Raw data | Compound Symmetry | Order | *F*_1, 13_ = 1.47 | 0.2463 |
|  |  |  | Place | *F*_1, 42_ = 5.19 | 0.0279 |
|  |  |  | Time | *F*_1, 42_ = 0.95 | 0.3365 |
|  |  |  | Place×Time | *F*_1, 42_ = 0.64 | 0.4279 |
| **Beta-C3 during N2 and N3** | Raw data | Compound Symmetry | Order | *F*_1, 13_ = 3.58 | 0.0810 |
|  |  |  | Place | *F*_1, 42_ = 3.88 | 0.0555 |
|  |  |  | Time | *F*_1, 42_ = 1.13 | 0.2939 |
|  |  |  | Place×Time | *F*_1, 42_ = 2.19 | 0.1462 |
| **Beta-O1 during N2 and N3** | Raw data | Compound Symmetry | Order | *F*_1, 13_ = 1.99 | 0.1813 |
|  |  |  | Place | *F*_1, 42_ = 5.73 | 0.0212 |
|  |  |  | Time | *F*_1, 42_ = 0.01 | 0.9093 |
|  |  |  | Place×Time | *F*_1, 42_ = 0.67 | 0.4177 |
| **Beta-O2 during N2 and N3** | Raw data | Compound Symmetry | Order | *F*_1, 13_ = 2.48 | 0.1391 |
|  |  |  | Place | *F*_1, 42_ = 2.05 | 0.1597 |
|  |  |  | Time | *F*_1, 42_ = 0.19 | 0.6665 |
|  |  |  | Place×Time | *F*_1, 42_ = 0.03 | 0.8729 |
| **SWA-F3 during N3** | Raw data | Compound Symmetry | Order | *F*_1, 13_ = 0.02 | 0.8900 |
|  |  |  | Place | *F*_1, 42_ = 1.44 | 0.2375 |
|  |  |  | Time | *F*_1, 42_ = 1.74 | 0.1947 |
|  |  |  | Place×Time | *F*_1, 42_ = 0.17 | 0.6842 |
| **SWA-F4 during N3** | Raw data | Compound Symmetry | Order | *F*_1, 13_ = 0.12 | 0.7343 |
|  |  |  | Place | *F*_1, 42_ = 1.28 | 0.2636 |
|  |  |  | Time | *F*_1, 42_ = 5.00 | 0.0308 |
|  |  |  | Place×Time | *F*_1, 42_ = 0.02 | 0.8956 |
| **SWA-C3 during N3** | Raw data | Compound Symmetry | Order | *F*_1, 13_ = 3.04 | 0.1049 |
|  |  |  | Place | *F*_1, 42_ = 1.08 | 0.3050 |
|  |  |  | Time | *F*_1, 42_ = 0.95 | 0.3365 |
|  |  |  | Place×Time | *F*_1, 42_ = 0.62 | 0.4355 |
| **SWA-C4 during N3** | Raw data | Compound Symmetry | Order | *F*_1, 13_ = 0.71 | 0.4134 |
|  |  |  | Place | *F*_1, 42_ = 0.03 | 0.8692 |
|  |  |  | Time | *F*_1, 42_ = 2.60 | 0.1142 |
|  |  |  | Place×Time | *F*_1, 42_ = 0.01 | 0.9277 |
| **SWA-O1 during N3** | Box-Cox | Autoregressive | Order | *F*_1, 21.5_ *=* 6.62 | 0.0175 |
|  |  |  | Place | *F*_1, 44.1_ *=* 0.02 | 0.8835 |
|  |  |  | Time | *F*_1, 20.8_ = 0.14 | 0.7159 |
|  |  |  | Place×Time | *F*_1, 48.3_ *=* 0.49 | 0.4886 |
| **SWA-O2 during N3** | Raw data | Unstructured | Order | *F*_1, 13.4_ = 6.66 | 0.0224 |
|  |  |  | Place | *F*_1, 13.1_ = 1.24 | 0.2851 |
|  |  |  | Time | *F*_1, 13.7_ *=* 4.59 | 0.0506 |
|  |  |  | Place×Time | *F*_1, 13.7_ *=* 0.19 | 0.6710 |
| **OSA-MA   “Initiation and maintenance of sleep”** | Raw data | Compound Symmetry | Order | *F*_1, 13_ = 3.94 | 0.0687 |
|  |  |  | Place | *F*_1, 42_ = 7.27 | 0.0101 |
|  |  |  | Time | *F*_1, 42_ = 0.97 | 0.3309 |
|  |  |  | Place×Time | *F*_1, 42_ = 1.60 | 0.2129 |
| **Temperature** | Box-Cox | Compound Symmetry | Order | *F*_1, 13_ = 2.27 | 0.1562 |
|  |  |  | Place | *F*_1, 42_ = 2.48 | 0.1232 |
|  |  |  | Time | *F*_1, 42_ = 0.05 | 0.8170 |
|  |  |  | Place×Time | *F*_1, 42_ = 0.01 | 0.9169 |
| **Temperature CV** | Box-Cox | Unstructured | Order | *F*_1, 13.2_ = 1.02 | 0.3311 |
|  |  |  | Place | *F*_1, 14_ = 6.96 | 0.0195 |
|  |  |  | Time | *F*_1, 11.8_ = 0.11 | 0.7470 |
|  |  |  | Place×Time | *F*_1, 13.5_ = 1.32 | 0.2708 |
| **Humidity CV** | Raw data | Compound Symmetry | Order | *F*_1, 13_ = 0.01 | 0.9366 |
|  |  |  | Place | *F*_1, 42_ = 51.20 | <0.0001 |
|  |  |  | Time | *F*_1, 42_ = 1.17 | 0.2853 |
|  |  |  | Place×Time | *F*_1, 42_ = 0.54 | 0.4647 |
| **Sound CV** | Box-Cox | Compound Symmetry | Order | *F*_1, 13_ = 0.09 | 0.7712 |
|  |  |  | Place | *F*_1, 42_ = 14.05 | 0.0005 |
|  |  |  | Time | *F*_1, 42_ = 1.53 | 0.2227 |
|  |  |  | Place×Time | *F*_1, 42_ = 0.04 | 0.8438 |
| Order indicates the Place (HSL or MSL) on the first and second nights and the third and fourth nights. Pre-exp represents on which night before the experiment the measurement was carried out. Place compares the HSL and MSL. Time compares the first nights (HSL and MSL) and the second nights (HSL and MSL).  ArI, arousal index; CV, coefficient of variation; HSL, Human Sleep Lab; MSL, Mobile Sleep Lab; SE, sleep efficiency; SL, sleep latency; SO, slow-oscillation; SWA, slow-wave activity; TST, total sleep time; WASO, wake after sleep onset; OSA-MA, Oguri-Shirakawa-Azumi Sleep Inventory, Middle-age and Aged version. | | | | | |
|  |  |  |  |  |  |
