# Supplementary material for: Mobile Sleep Lab: Comparison of polysomnographic parameters with a conventional sleep laboratory
Source: PLoS One. 2025 Jan 7;20(1):e0316579. doi: 10.1371/journal.pone.0316579 (PMC11706495; doi:10.1371/journal.pone.0316579)
Supplement: S4 Table — OSA-MA, Oguri-Shirakawa-Azumi Sleep Inventory, Middle-age and Aged version. (DOCX) [file pone.0316579.s012.docx]

**S4 Table. Results of statistical analyses of humidity, sound level, vibration level, self-reported sleep quality, and stage R latency, theta-wave activity in F3-M1 and C3-M1, and beta-wave activity in F4-M1and C4-M1 using nonparametric tests**.

| **Parameter** | ***F*-value** | ***P*-value** |
| --- | --- | --- |
| **Humidity** | *F*_(3)_ = 37.0000 | <0.0001 |
| **Sound level** | *F*_(3)_ = 4.2162 | 0.2390 |
| **Vibration level** | *F*_(3)_ = 1.0000 | 0.8013 |
| **OSA-MA   “Sleepiness on rising”** | *F*_(3)_ = 4.5115 | 0.2113 |
| **OSA-MA   “Frequent dreaming”** | *F*_(3)_ = 3.0541 | 0.3834 |
| **OSA-MA   “Refreshness”** | *F*_(3)_ = 2.9250 | 0.4033 |
| **OSA-MA   “Sleep length”** | *F*_(3)_ = 1.8132 | 0.6121 |
| **Stage R latency** | *F*_(3)_ = 3.7651 | 0.2880 |
| **Theta-F3 during N2 and N3** | *F*_(3)_ = 5.4800 | 0.1398 |
| **Theta-C3 during N2 and N3** | *F*_(3)_ = 1.8000 | 0.6149 |
| **Beta-F4 during N2 and N3** | *F*_(3)_ = 4.9200 | 0.1777 |
| **Beta-C4 during N2 and N3** | *F*_(3)_ = 4.9200 | 0.1777 |
| OSA-MA, Oguri-Shirakawa-Azumi Sleep Inventory, Middle-age and Aged version | | |
