# Supplementary material for: Mobile Sleep Lab: Comparison of polysomnographic parameters with a conventional sleep laboratory
Source: PLoS One. 2025 Jan 7;20(1):e0316579. doi: 10.1371/journal.pone.0316579 (PMC11706495; doi:10.1371/journal.pone.0316579)
Supplement: S5 Table — The values for S8 Fig, of the mean of the difference between the means of the two sleep variables (90% confidence intervals). (DOCX) [file pone.0316579.s013.docx]

**S5 Table. Equivalence of sleep variables assessed in the Human Sleep Lab (HSL) and Mobile Sleep Lab (MSL).**

| **Parameters** | **HSL 1^st^ & 2^nd^ night** | **MSL 1^st^ & 2^nd^ night** | **HSL 1^st^ & MSL 1^st^ night** | **HSL 2^nd^ & MSL 2^nd^ night** | **3^rd^ & 4^th^ night (HSL or MSL)** |
| --- | --- | --- | --- | --- | --- |
|  |  |  |  |  |  |
| **TST** | 12.9  (−4.9, 30.7) | −1.1  (−2.9, 0.7)* | 20.3  (5.0, 35.6) | 1.1  (−21.2, 23.5) | 3.5  (−16.8, 23.8) |
| **SE** | 12.9  (−4.9, 30.8) | −1.1  (−2.9, 0.7)* | 20.3  (4.9, 35.7) | 1.2  (−21.1, 23.6) | 3.7  (−16.7, 24.0) |
| **WASO** | −35.8  (−70.1, −1.4) | 8.0  (−10.2, 26.2) | −9.3  (−43.5, 24.9) | −2.1  (−33.5, 29.2) | −2.8  (−24.0, 18.4) |
| **%N1** | −2.3  (−32.3, 27.8) | 8.6  (−2.1, 19.3)* | −10.3  (−25.1, 4.5) | 13.0  (−4.9, 31.0) | 4.7  (−12.8, 22.2) |
| **%N2** | −0.8  (−8.5, 6.9)* | −0.9  (−10.1, 8.4)* | −6.1  (−13.8, 1.7)* | −6.2  (−13.9, 1.5)* | −1.8  (−9.6, 6.0)* |
| **%N3** | 3.8  (−3.0, 10.6)* | 1.1  (−7.0, 9.3)* | 10.4  (1.0, 19.8)* | 7.9  (0.5, 15.4)* | 2.0  (−4.7, 8.7)* |
| **%R** | −4.4  (−15.1, 6.3)* | −9.6  (−17.4, −1.8)* | 0.2  (−7.7, 8.0)* | −4.6  (−12.6, 3.4)* | −5.2  (−11.8, 1.4)* |
| **ArI** | 0.8  (−4.3, 6.0)* | −0.2  (−0.6, 0.2)* | 0.1  (−2.0, 2.3)* | −0.2  (−6.4, 6.1)* | 0.7  (−1.4, 2.8)* |
| **SL** | 9.0  (−15.1, 33.0) | 3.1  (−19.3, 25.5) | −9.3  (−43.5, 24.9) | −17.4  (−42.6, 7.9) | 0.5  (−26.7, 27.7) |
| **N1 latency** | 12.1  (−10.6, 34.8) | 3.1  (−19.3, 25.5) | −5.1  (−36.0, 25.9) | −16.3  (−41.6, 8.9) | 4.7  (−20.9, 30.2) |
| **N2 latency** | −16.0  (−37.3, 5.4) | 7.1  (−8.3, 22.5) | −22.0  (−49.1, 5.0) | 2.1  (−13.5, 17.6)* | −13.4  (−35.2, 8.5) |
| **N3 latency** | −2.2  (−4.9, 0.6)* | −1.4  (−11.0, 8.2)* | −2.1  (−10.3, 6.2)* | −0.1  (−1.6, 1.5)* | −4.0  (−9.1, 1.1)* |
| **Stage R latency** | 13.2  (−3.3, 29.7) | 12.1  (−4.8, 29.0) | −12.4  (−34.4, 9.6) | −5.9  (−22.5, 10.7) | 13.9  (−2.4, 30.2) |
| The values for S8 Fig, of the mean of the difference between the means of the two sleep variables (90% confidence intervals). Presence of asterisk (*) indicates equivalence.  ArI, arousal index; HSL, Human Sleep Lab; MSL, Mobile Sleep Lab; SE, sleep efficiency; SL, sleep latency; TST, total sleep time; WASO, wake after sleep onset. | | | | | |
