# Supplementary material for: Mobile Sleep Lab: Comparison of polysomnographic parameters with a conventional sleep laboratory
Source: PLoS One. 2025 Jan 7;20(1):e0316579. doi: 10.1371/journal.pone.0316579 (PMC11706495; doi:10.1371/journal.pone.0316579)
Supplement: S2 File — (DOCX) [file pone.0316579.s015.docx]

Terminology for each item in the S1 File

**ID:** ID used for anonymization purposes, with one participant corresponding to each ID

**age:** Age of participant (years)

**sex:** Sex of participant, male (M) or female (F)

**location:** Human Sleep Lab (HSL) or Mobile Sleep Lab (MSL)

**timepoint:** Number of measurements taken on the first and second night at each of HSL or MSL

**night**: Number of measurements on nights 1 to 4 for one participant at both HSL and MSL site

**OSA1:** Factor 1 of the Oguri-Shirakawa-Azumi Sleep Inventory, Middle-age and Aged version (OSA-MA), "sleepiness on rising"

**OSA2:** Factor 2 of the OSA-MA, "initiation and maintenance of sleep"

**OSA3:** Factor 3 of OSA-MA, "frequent dreaming"

**OSA4:** Factor 4 of OSA-MA, "refreshness"

**OSA5:** Factor 5 of OSA-MA, "sleep length"

**Fitbit_pre_X_sleeptime:** Total sleep time (TST) from Fitbit on the day of the experiment, where X is the number of nights before the experiment (1-3), and mean is the average of the three nights before the experiment (min)

**sound:** Mean value of the noise during 8 h of time in bed (TIB) on the experimental day (dB)

**vibration:** Mean value of vibration during 8 h of TIB on the experimental day (m/s^2^)

**temperature:** Mean room temperature during 8 h of TIB on the experimental day (°C)

**humidity:** Mean humidity during 8 h of TIB on the experimental day (%)

**sound_CV:** Mean coefficient of variation (CV) of the noise during 8 h of time in bed (TIB) on the experimental day (%)

**temperature_CV:** Mean CV of room temperature during 8 h of TIB on the experimental day (%)

**humidity_CV:** Mean CV of humidity during 8 h of TIB on the experimental day (%)

**PSG_TIB:** TIB measured by polysomnography (PSG) on the experimental day (min)

**PSG_TST:** Total sleep time (TST) measured using PSG on the experimental day (min)

**PSG_SE:** Sleep efficiency (SE) measured using PSG on the experimental day (%)

**PSG_SL:** Sleep latency (SL) measured by PSG on the experimental day (min)

**PSG_WASO:** Wake after sleep onset (WASO) measured using PSG on the experimental day (min)

**PSG_N1:** Duration of stage N1 as measured by PSG on experimental day (min).

**PSG_perN1:** Percentage of stage N1 to TST (%N1) measured by PSG on experimental day (%)

**PSG_N1latency:** Latency from light out to stage N1 as measured by PSG on the experimental day (min)

**PSG_N2:** Duration of stage N2 as measured by PSG on the experimental day (min)

**PSG_perN2:** %N2 measured by PSG on the experimental day (%)

**PSG_N2latency:** Latency from light out to stage N2 measured by PSG on the experimental day (min)

**PSG_N3:** Duration of stage N3 measured by PSG on experimental day (min)

**PSG_perN3:** %N3 measured by PSG on experimental day (%)

**PSG_N3latency:** Latency from light out to stage N3 measured by PSG on experimental day (min)

**PSG_R:** Duration of stage R measured by PSG on experimental day (min)

**PSG_perR:** %R as measured by PSG on the experimental day (%)

**PSG_stageRlatency:** Stage R latency as measured by PSG on the experimental day (min)

**PSG_arousal:** Number of arousals measured by PSG on the experimental day

**PSG_ArI:** Arousal index (ArI) measured by PSG on experimental day (/h)

**SWA_F3_N2N3:** Slow-wave activity (SWA) in F3-M2 derivation at stages N2 and N3 [log(μV^2^/Hz)]

**SWA_F4_N2N3:** SWA in F4-M1 derivation at stages N2 and N3 [log(μV^2^/Hz)]

**SWA_C3_N2N3:** SWA in C3-M2 derivation at stages N2 and N3 [log(μV^2^/Hz)]

**SWA_C4_N2N3:** SWA in C4-M1 derivation at stages N2 and N3 derivation [log(μV^2^/Hz)]

**SWA_O1_N2N3:** SWA in O1-M2 derivation at stages N2 and N3 [log(μV^2^/Hz)]

**SWA_O2_N2N3:** SWA in O2-M1 derivation at stages N2 and N3 [log(μV^2^/Hz)]

**SWE_X_N2N3:** Slow-wave energy (SWE) in the X derivation at stages N2 and N3 [log(μV^2^/Hz)]

**Delta_X_N2N3**: Delta wave activity in the X deviation at stages N2 and N3 [log(μV^2^/Hz)]

**SO_X_N2N3:** Slow oscillation in the X deviation at stage N2 and N3 [log(μV^2^/Hz)]

**Theta_X_N2N3:** Theta-wave activity in the X deviation at stage N2 and N3 [log(μV^2^/Hz)]

**Alpha_X_N2N3:** Alpha-wave activity in the X deviation at stage N2 and N3 [log(μV^2^/Hz)]

**Sigma_X_N2N3:** Sigma-wave activity in the X deviation at stage N2 and N3 [log(μV^2^/Hz)]

**Beta_X_N2N3:** Beta-wave activity in the X deviation at stage N2 and N3 [log(μV^2^/Hz)]

**SWA_X_N3:** SWA in X derivation at stages N3 [log(μV^2^/Hz)]
