# Supplementary material for: Mobile Sleep Lab: Comparison of polysomnographic parameters with a conventional sleep laboratory
Source: PLoS One. 2025 Jan 7;20(1):e0316579. doi: 10.1371/journal.pone.0316579 (PMC11706495; doi:10.1371/journal.pone.0316579)
Supplement: Attachment — Submitted filename: Response_to_Reviewer.docx [file pone.0316579.s018.docx]

**Response to Reviewers**

**Reviewers' comments:**

**Editor #1_1: *On my end, the link to Zenodo did not work (I did not have the appropriate privileges for the private link). I encourage you to make it public or ensure we can check the availability of the data. Please also add a statement in the main text that the processed data are available as supplemental information and the raw data are provided without restriction on zenodo (with the correct link). This will facilitate data re-use.***

**Response:** Thank you for your comment. We are sorry for the inappropriate link, and we have published the data on Zenodo and modified the link.

We have added the following sentence on page 10, lines 211-213: “The processed data are available as supplemental information and the raw data are provided without restriction on Zenodo (<https://doi.org/10.5281/zenodo.14020530>) [27].” We have also added the following sentence to the Supporting Information, page 38, lines 932-933: “The raw data are available without restriction on Zenodo (<https://doi.org/10.5281/zenodo.14020530>) [27].” We have added reference number 27 for the published data: “Suzuki C, Suzuki Y, Abe T, Kanbayashi T, Fukusumi S, Kokubo T et al. Mobile Sleep Lab: Comparison of polysomnographic parameters with a conventional sleep laboratory [Data set]. Zenodo. 2024; <https://doi.org/10.5281/zenodo.14020530>.”

　We also found an error in one record of the sleep staging data used for frequency analysis. The S3 File and the frequency analysis results in the manuscript, S1 File (highlighted in yellow), and S3 Table (highlighted in blue) have been modified. The non-parametric tests results of the frequency analysis and the summary of the results and discussion are unchanged.

**Journal requirement_1: *Please review your reference list to ensure that it is complete and correct. If you have cited papers that have been retracted, please include the rationale for doing so in the manuscript text, or remove these references and replace them with relevant current references. Any changes to the reference list should be mentioned in the rebuttal letter that accompanies your revised manuscript. If you need to cite a retracted article, indicate the article’s retracted status in the References list and also include a citation and full reference for the retraction notice.***

**Response:** Thank you for your comment and information. We rechecked the reference list to see whether it is complete and correct. We cited the first guideline for the 10-20 system in the previous manuscript. However, since there has been a subsequent revision of the guideline, we have replaced the cited references with the revised version. We replaced reference 26 as follows: Klem GH, Lüders HO, Jasper HH, Elger C. The ten-twenty electrode system of the International Federation. The International Federation of Clinical Neurophysiology. Electroencephalogr Clin Neurophysiol Suppl. 1999;52: 3-6.

**Reviewer #1_1: *Thank you for the thorough revisions you have made to the manuscript. I have a few remaining comments regarding the statistical analyses that need to be addressed:***

***- On page 22 (lines 511-520), the authors mentioned that spectral power analyses were performed without corrections for multiple comparisons. I recommend applying appropriate corrections for multiple comparisons across all the analyses and reporting results that survive these corrections.***

**Response:** Thank you for your confirmation. We apologize – the statement that no correction is made was incorrect, since Bonferonni correction is made at each electrode. Since the *p*-value results in the manuscript are the results after Bonferroni correction, the results remain the same. We did not correct the inter-electrode p-values because we did not evaluate the topographic distribution of the power values in the frequency analysis.

The text was modified as follows on page 23, lines 544-545: “At the same time, statistical analysis was performed 54 times for the frequency index.” by deleting the phrase *“and each electrode with no correction”*

**Reviewer #1_2: *Similarly, regarding the new analyses conducted on sound level, there was an effect of time during the 8-hour period in the mobile sleep lab (MSL), with higher sound level observed in the first hour compared to later hours in the second half of the night. Were these analyses also corrected for multiple comparisons? Please clarify this.***

**Response:** Thank you for your confirmation. Bonferroni correction for multiple comparisons was already used in the sound level analysis in the previous manuscript. We did not describe this fact, so we added the sentence in the Methods on page 15, lines 321-322: “For multiple comparisons, Bonferroni correction was used.”

In relation to this comment, we have reviewed the analysis and have changed our response for the previous reviewer #1_5_11's comment: ***The authors extensively discussed the potential causes for the difference in the level of humidity in the two labs. Based on Fig. S3, it seems that the averaged level of humidity is in the normal range (around 50-60%) or a bit higher in the conventional lab. Does the level of humidity fluctuate throughout the night? If yes, does this fluctuation differ between the 2 labs? (same question for temperature and noise). For noise, if it is higher during the beginning of the night (due for example to lower external soundproofing in the bus), it can affect the first part of the night which is richer in SWA.*** In the previous manuscript, we evaluated temperature, humidity, and sound level fluctuations overnight by calculating the hourly coefficient of variations (CVs), but we thought that calculating CVs over 8 h and quantitatively evaluating them would be a more appropriate way to compare between the HSL and MSL, rather than comparing hourly CVs. Therefore, we recalculated the CVs from 8-h recordings. CV results and explanations were added to the S1 File (highlighted in green), S3 Table (highlighted in yellow), and S2 File (highlighted in yellow), respectively. The results showed that the CVs for temperature and humidity were higher in the MSL, while noise fluctuations were lower in the MSL compared to the HSL. Related to this change, pannels in the S4 Figure for each hourly CV evaluation were removed. We have modified the text in the Methods from page 9, line 191 to page 10, line 194: “We analyzed the temperature, humidity, sound, vibration, and the coefficient of variation (CV) of each parameter only during the 8 h of nighttime sleep. We calculated the average temperature, humidity, and sound in each hour separately, and the CV in each hour separately.”. We have added following the results at page 15, line 343 to page 16, line 349: “There were main effects of place in CVs on temperature, humidity, and sound between the two laboratories (*F*_1, 14_ = 6.96, *p* = 0.020, *F*_1, 42_ = 51.20, *p* <0.0001, *F*_1, 42_ = 14.05, *p* <0.001, respectively; S3 Table). CVs of temperature and humidity showed significant increases in the MSL compared with the HSL (*t_14_* = −2.64, *p* = 0.020, *t_42_* = −7.16, *p* <0.0001, respectively). On the contrary, the CV of sound level significantly decreased in the MSL compared to the HSL (*t*_42_ = 3.75, *p* <0.001).” The discussion is unchanged.

We apologize for our mistake; in the previous manuscript, the statistical results in the hourly evaluation of environmental factors in the manscript were only sound results. Furthermore, the statistical results in the S4 Figure were also insufficient and difficult to understand. We have added hourly averaged temperature and humidity results in the manuscript, and all statistical results for temperature, humidity, and sound levels are now shown in S4 Figure using asterisks. We have added the following results on page 16, lines 350-358: “The average was calculated every hour to examine the effects of changes in environmental measurements on sleep over time (S4 Fig). In temperature, a main effect of time was found for MSL (first night: *F*_(7)_ = 25.03, *p* <0.001; second night: *F*_(7)_ = 17.5, *p* = 0.014). No significant differences by time for both MSL nights were found in post-hoc tests (all *ps* >0.05, S4 Fig. A). In humidity, a main effect of time was found for HSL (first night: *F*_(7)_ = 45.4, *p* <0.0001; second night: *F*_(7)_ = 22.1, *p* = 0.002). Post-hoc tests revealed a significant increase in humidity at hour 7 versus hour 3 for the first night of HSL (*Z* = −3.351, *p* = 0.023, S4 Fig. B). There were no significant differences in post-hoc tests on the second night at the HSL (all *ps* >0.05).”.

**Reviewer #1_3: *Additionally, authors reported a correlation between %N3 and subjective sleep initiation and maintenance. Did they test whether %N3 also correlates with sound level? Given that the authors hypothesize that the higher sound level in the first hour may explain the reduced %N3 in the MSL, it would be useful to examine this relationship. If a correlation exists between sound level and %N3, it could further support the interpretation that the reduced %N3 is attributable to environmental factors (e.g., sound) rather than stress. This would strengthen the conclusion that sleep parameters in the MSL are comparable to those obtained with conventional polysomnography, if sound levels are adequately controlled throughout the night.***

**Response:** Thank you for your advice. To investigate whether the environmental factor, sound level, was associated with a decrease in %N3, we evaluated the correlation between %N3 and sound level using hourly data from N = 472 except for intervals where sleep was not occurring and sound level failed to be measured. Since %N3 was not correlated with sound level (*r_s_* = 0.10, *p* = 0.112 in the HSL; *r_s_* = 0.03, *p* = 0.665 in the MSL, S5 Figure), the decrement in %N3 in the MSL may not be explained by the influence of the sound level. We have included the following sentences in the Methods on page 15, lines 322-326: “As we found a significant increase in averaged sound in the first hour of MSL recording, we calculated the correlation between the %N3 and sound level in each hour from N = 472 data. Data intervals were removed where sleep was not occurring and sound level failed to be measured. Spearman’s correlation coefficient was used to evaluate whether the %N3 decrement was related to sound level.” We added the following descriptions to the Results on page 16, lines 365-369: “To evaluate whether sound level increment in the first hour related to %N3 decrement in the MSL, correlations between hourly averaged sound level and hourly averaged %N3 were evaluated for the HSL and MSL. No significant correlation was found between sound levels in the HSL and MSL, respectively (*r_s_* = 0.10, *p* = 0.112, *r_s_* = 0.03, *p* = 0.665, S5 Fig. A and B). ” We have added the following sentence on page 22, lines 512-514: “However, there was no correlation between %N3 and sound level, so the decrease in %N3 at the MSL may not be explained by the environmental sound factor.” We have deleted the following sentence from the Discussion: *“That is, sound level fluctuation might partly relate to the reduction %N3 in MSL.”*

**Reviewer #1_4: *Finally, there is a minor correction needed in Figure 2: the number of participants is noted as N = 16, but the final sample includes 15 participants. Please adjust or remove this notation accordingly.***

**Response:** Thank you for your confirmation. In Fig 2A, N = 16 on the left side was corrected to N = 15. In addition, the number in each group (N = 7 and N = 8) is shown on the right side of Fig 2A. The figure caption on page 8, lines 145-147 was corrected as follows: “The participants were randomly allocated to two groups. The first group (n = 7) underwent sleep measurement at the HSL for the first two nights, and at the MSL for the next two nights; this sequence was reversed for the second group (n = 8).”
