# Supplementary material for: Mobile Sleep Lab: Comparison of polysomnographic parameters with a conventional sleep laboratory
Source: PLoS One. 2025 Jan 7;20(1):e0316579. doi: 10.1371/journal.pone.0316579 (PMC11706495; doi:10.1371/journal.pone.0316579)
Supplement: Attachment — Submitted filename: Response_to_Reviewer.docx [file pone.0316579.s019.docx]

**Response to Reviewers**

**Reviewers' comments:**

**Journal requirement_1: *Please review your reference list to ensure that it is complete and correct. If you have cited papers that have been retracted, please include the rationale for doing so in the manuscript text, or remove these references and replace them with relevant current references. Any changes to the reference list should be mentioned in the rebuttal letter that accompanies your revised manuscript. If you need to cite a retracted article, indicate the article’s retracted status in the References list and also include a citation and full reference for the retraction notice.***

**Response:** Thank you for your comments and suggestions. We have reviewed and corrected the reference list. There are no retracted articles.

**Reviewer #1_1: *Thank you for addressing the previous comments and for revising the manuscript. However, I would like to ask for further clarification regarding your analysis of environmental factors, especially the sound level.***

***In your response, you mentioned: “In the previous manuscript, we evaluated temperature, humidity, and sound level fluctuations overnight by calculating the hourly coefficient of variations (CVs), but we thought that calculating CVs over 8 h and quantitatively evaluating them would be a more appropriate way to compare between the HSL and MSL, rather than comparing hourly CVs.” While this methodological change is acknowledged, it lacks a clear justification. Could you please elaborate on why you believe this approach is more appropriate and provide a stronger rationale for this methodological shift?***

**Response:** Thank you for your confirmation. We chose to evaluate the CVs during the 8-h sleep period because we believe that analyzing CVs over an 8-h period provides a more comprehensive reflection of overall variability during sleep compared to examining hourly changes in CVs. We considered that this analysis would more appropriately address your initial comment: "Does the level of humidity fluctuate throughout the night?" In contrast, we thought that the analysis of hourly changes in average values was suitable for addressing your comment. Therefore, we kept the analysis of averages as it was.

***Additionally, in lines 324–326, you state: “As we found a significant increase in averaged sound in the first hour of MSL recording, we calculated the correlation between the %N3 and sound level in each hour from N = 472 data.” This implies that you still performed an hourly analysis. However, later in the Results section, the correlation is described as being calculated between hourly averaged sound levels and hourly averaged %N3, with no apparent differences between the MSL and HSL. Given that the sound level was significantly higher during the first hour of recording in the MSL, I would recommend performing a focused analysis of the correlation between %N3 and sound level specifically during the first hour of the night, rather than averaging this measure across the entire night. This approach would directly address the potential influence of the elevated sound levels observed at the start of the recording.***

***If you choose not to follow this recommendation, I kindly ask that you provide a detailed rationale for why the averaged measures across the night were deemed more appropriate for this analysis, despite the observed increase in sound during the first hour.***

**Response:** Thank you for your valuable feedback about focusing on the first hour and seeking the relationship between %N3 and sound level. We analyzed it according to your suggestion. No correlation was seen between %N3 and noise at MSL. Thus, increasing noise during the first hour of sleep recording at the MSL may not be associated with a decrease in %N3 at the MSL. However, surprisingly, a positive correlation was found between %N3 and sound level in the first hour at the HSL. That is, %N3 increased with increasing sound level during the first hour in the HSL. The reason for this remains unclear.

According to the suggested analysis, we added the following to the methods on page 15, lines 353-359: “As in the second night of MSL, the average hourly sound level at 1 h after lights out was higher than at 4 and 6–8 h, so we focused on the correlation between %N3 and sound level during the first hour. We predicted that the increase in sound level would be related to the decrease in %N3 in the MSL. The first and second nights were combined with the corresponding HSL and MSL to obtain the respective correlation coefficients between %N3 and sound level.”

We have added the following to the results on page 17, lines 402-407: “As an increase in sound level was observed at 1 h after lights out compared to 4 and 6–8 h in the second night of the MSL, the correlation between %N3 and sound level was examined focusing on the first hour after lights out. The results showed a positive correlation between %N3 and sound level in the HSL (*r_s_* = 0.42, *p* = 0.023, S5 Fig. C) and no correlation between %N3 and sound level in the MSL (*r_s_* = 0.25, *p* = 0.187, S5 Fig. D).”

We have added the following discussion from page 22, line 550 to page 23, line 555: “As an increased sound level might influence the decrease in %N3 in the MSL, we performed a correlation analysis. Contrary to our expectation, %N3 increased with increasing sound level during the first hour after light out in the HSL. The reason for this remains unclear. However, there was no correlation between %N3 and sound level in the MSL, so the environmental sound factor cannot explain the decrease in %N3 in the MSL.”

We have added Supplemental Information for scatter plots of %N3 and sound level in the first hour to S5 Fig. C and D.
